# Supplementary material for: The CD8+ and CD4+ T Cell Immunogen Atlas of Zika Virus Reveals E, NS1 and NS4 Proteins as the Vaccine Targets
Source: Viruses. 2022 Oct 25;14(11):2332. doi: 10.3390/v14112332 (PMC9696057; doi:10.3390/v14112332)
Supplement: Supplementary file 1 [file viruses-14-02332-s001.zip › viruses-1956334-supplementary.pdf]

Supplementary Materials

Figure S1. Peptides immune-thermogram analysis of CD8+ /CD4+ T-cell in H-2b and H-2d mice.

Wild-type BALB/c (H-2d) mice were infected with 104 FFU of ZIKV, splenocytes were harvested at 14 d.p.i. and stimulated with above-positive peptides to assess cytokines production by ICS. The percentages and heat map analysis of IFN-γ, TNF-α and IL-2 produced in CD8+ /CD4+ T cells in H-2b and H-2d mice (n=3 per peptide). Dashed lines beyond red are strongly positive.

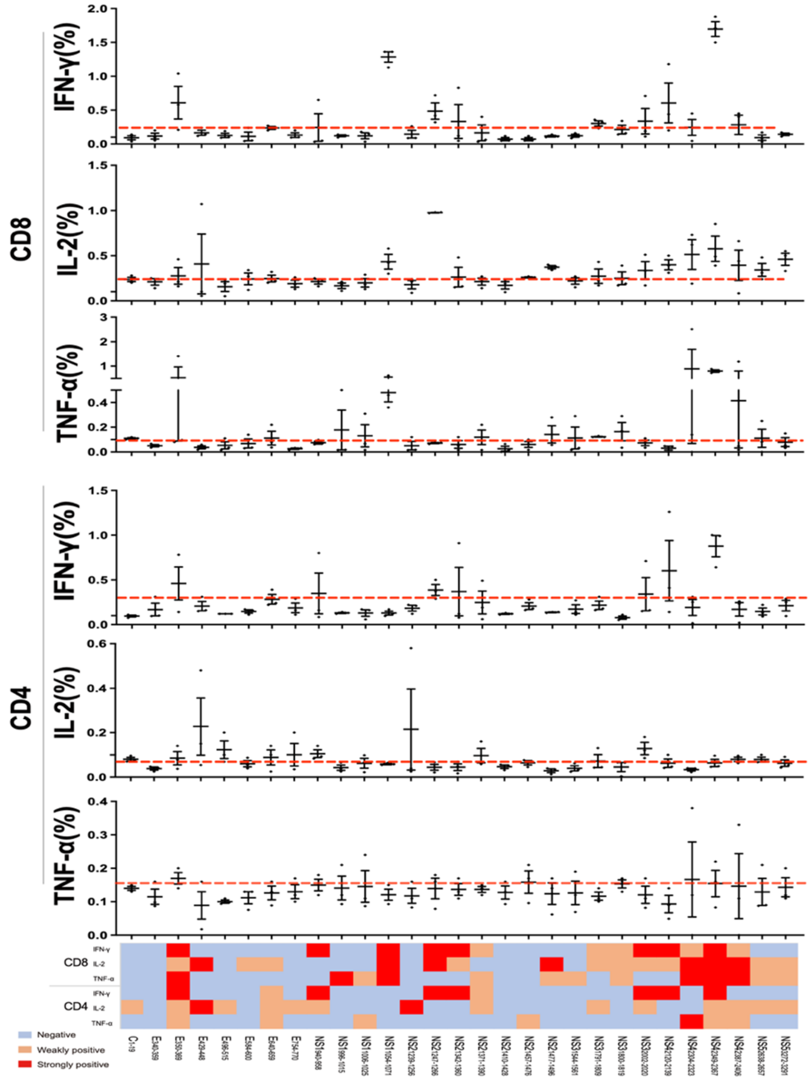

Table S1. 2-D matrix pool

Matrix1

	1-1	1-2	1-3	1-4	1-5	1-6	1-7	1-8	1-9	1-10	1-11	1-12
1-A	**ZV-C1**	**ZV-C2**	**ZV-C3**	**ZV-C4**	**ZV-C5**	**ZV-C6**	**ZV-C7**	**ZV-C8**	**ZV-C9**	**ZV-C10**	**ZV-C11**	**ZV-C12**
1-B	**ZV-C13**	**ZV-PR1***	**ZV-PR2**	**ZV-PR3**	**ZV-PR4**	**ZV-PR5**	**ZV-PR6**	**ZV-PR7**	**ZV-PR8**	**ZV-PR9**	**ZV-PR10**	**ZV-PR11**
1-C	**ZV-PR12**	**ZV-PR13**	**ZV-PR14**	**ZV-PR15**	**ZV-PR16**	**ZV-PR17**	**ZV-E1**	**ZV-E2**	**ZV-E3**	**ZV-E4**	**ZV-E5**	**ZV-E6**
1-D	**ZV-E7**	**ZV-E8**	**ZV-E9**	**ZV-E10**	**ZV-E11**	**ZV-E12**	**ZV-E13**	**ZV-E14**	**ZV-E15**	**ZV-E16**	**ZV-E17**	**ZV-E18**
1-E	**ZV-E19**	**ZV-E20**	**ZV-E21**	**ZV-E22**	**ZV-E23**	**ZV-E24**	**ZV-E25**	**ZV-E26**	**ZV-E27**	**ZV-E28**	**ZV-E29**	**ZV-E30**
1-F	**ZV-E31**	**ZV-E32**	**ZV-E33**	**ZV-E34**	**ZV-E35**	**ZV-E36**	**ZV-E37**	**ZV-E38**	**ZV-E39**	**ZV-E40**	**ZV-E41**	**ZV-E42**
1-G	**ZV-E43**	**ZV-E44**	**ZV-E45**	**ZV-E46**	**ZV-E47**	**ZV-E48**	**ZV-E49**	**ZV-E50**	**ZV-E51**	**ZV-E52**	**ZV-E53**	**ZV-NS1-1**

Matrix 2

	2-1	2-2	2-3	2-4	2-5	2-6	2-7	2-8	2-9	2-10	2-11	2-12
2-A	**ZV-NS1-14**	**ZV-NS1-15**	**ZV-NS1-16**	**ZV-NS1-17**	**ZV-NS1-18**	**ZV-NS1-19**	**ZV-NS1-20**	**ZV-NS1-21**	**ZV-NS1-22**	**ZV-NS1-23**	**ZV-NS1-24**	**ZV-NS1-25**
2-B	**ZV-NS1-26**	**ZV-NS1-27**	**ZV-NS1-28**	**ZV-NS1-29**	**ZV-NS1-30**	**ZV-NS1-31**	**ZV-NS1-32**	**ZV-NS1-33**	**ZV-NS1-34**	**ZV-NS1-35**	**ZV-NS1-36**	**ZV-NS1-37**
2-C	**ZV-NS1-38**	**ZV-NS2-1**	**ZV-NS2-2**	**ZV-NS2-3**	**ZV-NS2-4**	**ZV-NS2-5**	**ZV-NS2-6**	**ZV-NS2-7**	**ZV-NS2-8**	**ZV-NS2-9**	**ZV-NS2-10**	**ZV-NS2-11**
2-D	**ZV-NS2-12**	**ZV-NS2-13**	**ZV-NS2-14**	**ZV-NS2-15**	**ZV-NS2-16**	**ZV-NS2-17**	**ZV-NS2-18**	**ZV-NS2-19**	**ZV-NS2-20**	**ZV-NS2-21**	**ZV-NS2-22**	**ZV-NS2-23**
2-E	**ZV-NS2-24**	**ZV-NS2-25**	**ZV-NS2-26**	**ZV-NS2-27**	**ZV-NS2-28**	**ZV-NS2-29**	**ZV-NS2-30**	**ZV-NS2-31**	**ZV-NS2-32**	**ZV-NS2-33**	**ZV-NS2-34**	**ZV-NS2-35**
2-F	**ZV-NS2-36**	**ZV-NS2-37**	**ZV-NS3-1**	**ZV-NS3-2**	**ZV-NS3-3**	**ZV-NS3-4**	**ZV-NS3-5**	**ZV-NS3-6**	**ZV-NS3-7**	**ZV-NS3-8**	**ZV-NS3-9**	**ZV-NS3-10**

2-G	ZV-NS3-11	ZV-NS3-12	ZV-NS3-13	ZV-NS3-14	ZV-NS3-15	ZV-NS3-16	ZV-NS3-17	ZV-NS3-18	ZV-NS3-19	ZV-NS3-20	ZV-NS3-21	ZV-NS3-22

Matrix 3

	3-1	3-2	3-3	3-4	3-5	3-6	3-7	3-8	3-9	3-10	3-11	3-12
3-A	ZV-NS3-35	ZV-NS3-36	ZV-NS3-37	ZV-NS3-38	ZV-NS3-39	ZV-NS3-40	ZV-NS3-41	ZV-NS3-42	ZV-NS3-43	ZV-NS3-44	ZV-NS3-45	ZV-NS3-46
3-B	ZV-NS3-47	ZV-NS3-48	ZV-NS3-49	ZV-NS3-50	ZV-NS3-51	ZV-NS3-52	ZV-NS3-53	ZV-NS3-54	ZV-NS3-55	ZV-NS3-56	ZV-NS3-57	ZV-NS3-58
3-C	ZV-NS3-59	ZV-NS3-60	ZV-NS3-61	ZV-NS3-62	ZV-NS3-63	ZV-NS3-64	ZV-NS3-65	ZV-NS3-66	ZV-NS4-1	ZV-NS4-2	ZV-NS4-3	ZV-NS4-4
3-D	ZV-NS4-5	ZV-NS4-6	ZV-NS4-7	ZV-NS4-8	ZV-NS4-9	ZV-NS4-10	ZV-NS4-11	ZV-NS4-12	ZV-NS4-13	ZV-NS4-14	ZV-NS4-15	ZV-NS4-16
3-E	ZV-NS4-17	ZV-NS4-18	ZV-NS4-19	ZV-NS4-20	ZV-NS4-21	ZV-NS4-22	ZV-NS4-23	ZV-NS4-24	ZV-NS4-25	ZV-NS4-26	ZV-NS4-27	ZV-NS4-28
3-F	ZV-NS4-29	ZV-NS4-30	ZV-NS4-31	ZV-NS4-32	ZV-NS4-33	ZV-NS4-34	ZV-NS4-35	ZV-NS4-36	ZV-NS4-37	ZV-NS4-38	ZV-NS4-39	ZV-NS4-40
3-G	ZV-NS4-41	ZV-NS5-1	ZV-NS5-2	ZV-NS5-3	ZV-NS5-4	ZV-NS5-5	ZV-NS5-6	ZV-NS5-7	ZV-NS5-8	ZV-NS5-9	ZV-NS5-10	ZV-NS5-11

Matrix 4

	4-1	4-2	4-3	4-4	4-5	4-6	4-7	4-8	4-9	4-10	4-11	4-12
4-A	ZV-NS5-24	ZV-NS5-25	ZV-NS5-26	ZV-NS5-27	ZV-NS5-28	ZV-NS5-29	ZV-NS5-30	ZV-NS5-31	ZV-NS5-32	ZV-NS5-33	ZV-NS5-34	ZV-NS5-35
4-B	ZV-NS5-36	ZV-NS5-37	ZV-NS5-38	ZV-NS5-39	ZV-NS5-40	ZV-NS5-41	ZV-NS5-42	ZV-NS5-43	ZV-NS5-44	ZV-NS5-45	ZV-NS5-46	ZV-NS5-47
4-C	ZV-NS5-48	ZV-NS5-49	ZV-NS5-50	ZV-NS5-51	ZV-NS5-52	ZV-NS5-53	ZV-NS5-54	ZV-NS5-55	ZV-NS5-56	ZV-NS5-57	ZV-NS5-58	ZV-NS5-59
4-D	ZV-NS5-60	ZV-NS5-61	ZV-NS5-62	ZV-NS5-63	ZV-NS5-64	ZV-NS5-65	ZV-NS5-66	ZV-NS5-67	ZV-NS5-68	ZV-NS5-69	ZV-NS5-70	ZV-NS5-71
4-E	ZV-NS5-72	ZV-NS5-73	ZV-NS5-74	ZV-NS5-75	ZV-NS5-76	ZV-NS5-77	ZV-NS5-78	ZV-NS5-79	ZV-NS5-80	ZV-NS5-81	ZV-NS5-82	ZV-NS5-83
4-F	ZV-NS5-84	ZV-NS5-85	ZV-NS5-86	ZV-NS5-87	ZV-NS5-88	ZV-NS5-89	ZV-NS5-90	ZV-NS5-91	ZV-NS5-92	ZV-NS5-93	ZV-NS5-94	ZV-NS5-95

4-G	**ZV-NS5-96**	**ZV-NS5-97**	**ZV-NS5-98**	**ZV-NS5-99**								

*2-D matrix pools (X-axis:1-1 to 4-12, Y-axis:1-A to 4-G): if peptide pool1-2 and pool1-B detected positive, the cross peptide of
